# An intensified systemic trafficking of bone marrow-derived stem/progenitor cells in patients with pancreatic cancer

**DOI:** 10.1111/jcmm.12065

**Published:** 2013-05-15

**Authors:** Teresa Starzyńska, Krzysztof Dąbkowski, Wojciech Błogowski, Ewa Zuba-Surma, Marta Budkowska, Daria Sałata, Barbara Dołęgowska, Wojciech Marlicz, Jerzy Lubikowski, Mariusz Z Ratajczak

**Affiliations:** a Department of Gastroenterology, Pomeranian Medical UniversitySzczecin, Poland; b Department of Cell Biology, Faculty of Biochemistry, Biophysics and Biotechnology, Jagiellonian UniversityCracow, Poland; c Department of Laboratory Diagnostics and Molecular Medicine, Pomeranian Medical UniversitySzczecin, Poland; d Division of Hepatobiliary Surgery and Liver Transplantation, Marie Curie HospitalSzczecin, Poland; e Department of Physiology, Pomeranian Medical UniversitySzczecin, Poland; f Stem Cell Institute at the James Graham Brown Cancer Center, University of LouisvilleLouisville, KY, USA

**Keywords:** bone marrow-derived stem cells, complement cascade, growth/inhibitory factors, pancreatic cancer, SDF-1, S1P

## Abstract

Various experimental studies indicate potential involvement of bone marrow (BM)-derived stem cells (SCs) in malignancy development and progression. In this study, we comprehensively analysed systemic trafficking of various populations of BM-derived SCs (BMSCs), *i.e*., mesenchymal, haematopoietic, endothelial stem/progenitor cells (MSCs, HSCs, EPCs respectively), and of recently discovered population of very small embryonic/epiblast-like SCs (VSELs) in pancreatic cancer patients. Circulating CD133^+^/Lin^−^/CD45^−^/CD34^+^ cells enriched for HSCs, CD105^+^/STRO-1^+^/CD45^−^ cells enriched for MSCs, CD34^+^/KDR^+^/CD31^+^/CD45^−^ cells enriched for EPCs and small CXCR4^+^CD34^+^CD133^+^ subsets of Lin^−^CD45^−^ cells that correspond to VSELs were enumerated and sorted from blood samples derived from 29 patients with pancreatic cancer, and 19 healthy controls. In addition, plasma levels of stromal-derived factor-1 (SDF-1), growth/inhibitory factors and sphingosine-1-phosphate (S1P; chemoattractants for SCs), as well as, of complement cascade (CC) molecules (C3a, C5a and C5b-9/membrane attack complex – MAC) were measured. Higher numbers of circulating VSELs and MSCs were detected in pancreatic cancer patients (*P* < 0.05 and 0.01 respectively). This trafficking of BMSCs was associated with significantly elevated C5a (*P* < 0.05) and C5b-9/MAC (*P* < 0.005) levels together with S1P concentrations detected in plasma of cancer patients, and seemed to be executed in a SDF-1 independent manner. In conclusion, we demonstrated that in patients with pancreatic cancer, intensified peripheral trafficking of selected populations of BMSCs occurs. This phenomenon seems to correlate with systemic activation of the CC, hepatocyte growth factor and S1P levels. In contrast to previous studies, we demonstrate herein that systemic SDF-1 levels do not seem to be linked with increased mobilization of stem cells in patients with pancreatic cancer.

## Introduction

Pancreatic cancer constantly remains a leading cause of mortality among western societies. This grieving tendency continues mainly because of constant clinical difficulties in its early detection, as well as, limited treatment opportunities for advanced disease, presented by vast majority of affected individuals upon diagnosis. In addition, exact molecular mechanisms responsible for pancreatic malignancy development seem to be poorly understood 1, 2.

Within the last decades the ‘cancer stem cell’ hypothesis has been proposed, which postulates a novel concept of hierarchical organization of cells, in which ‘primitive’ cancer-initiating SCs were believed to be responsible for development and growth of tumours, and promotion of their metastatic spread (reviewed in 3). So far, results derived from analysis of experimental animal models or cell lines of breast or colorectal malignancies delivered some evidence supporting this concept 4, 5. Nevertheless, a breakthrough discovery was made by Houghton and colleagues 6, who demonstrated that gastric cancer may originate from SCs derived from the BM. As was further confirmed in experimental animals, BMSCs may participate in both – the development and progression of gastrointestinal tumours 6–8. Unfortunately, these observations have never been verified in clinical setting.

In the BM environment, several populations of SCs may be found, and these include (*i*) HSCs responsible for repopulation of blood cells, (*ii*) MSCs participating in regeneration of various organs (mainly derived from mesenchyme), and/or (*iii*) EPCs that may contribute to neovascularization. However, recently a novel population of SCs has been identified in murine and human BM, that is a population of VSELs 9, 10. From the morphological point of view, this unique group of very small (4–7 μm) Lin^−^CD45^−^CD133^+^ SCs resembles developmentally ‘primitive’ cells, and expresses genetic markers characteristic for embryonic/pluripotent SCs, such as Oct-4, Nanog, SSEA-4 and Rex1. In addition, VSELs remain quiescent in dormant state in tissues due to specific reprogramming of a somatic genomic imprinting, and in comparison to other populations of SCs possess around 10–200 times much higher expression of genes necessary for trans-differentiation into tissues/organs originating from all three germ layers (including GI tract) 9–11. Therefore, their powerful molecular potential and characteristics makes them an ideal potential candidate population of SCs that may be responsible for initiation of malignancy development in human beings and/or supporting growing tumour by supplying cells involved in its neovascularization and stromalization 12.

Taking all these facts into consideration, in this study, we wanted to translate these previous observations, and comprehensively analyse whether intensified peripheral trafficking of various populations of BMSCs is observed in patients with pancreatic cancer. In addition, we decided to examine (*i*)systemic levels of an established SCs chemoattractant – stromal-derived factor-1 (SDF-1), (*ii*)a wide panel of growth/inhibitory factors [vascular/endothelial, epidermal, hepatocyte, insulin-like growth factors (VEGF, EGF, HGF and IGF respectively) and leukaemia inhibitory factor-LIF], that could be responsible for observed alterations in peripheral circulation of BMSCs, as well as, (*iii*)whether systemic trafficking of BMSCs in pancreatic cancer patients is associated with plasma levels of molecules released during activation of CC (C3a, C5a and C5b-9/MAC) and sphingosine-1-phosphate (S1P), as proposed in the recent studies 13, 14. Our hypothesis was that in patients with pancreatic cancer, intensified systemic trafficking of various BMSCs populations (mainly VSELs and MSCs) occurs in peripheral blood (PB), and is associated with increased levels of immunomodulatory compounds, such as complement-derived molecules and/or S1P.

## Materials and methods

### Patients and blood samples

A total of 29 individuals in a general good state with freshly diagnosed pancreatic cancer, and 19 healthy controls were included in the study. All patients recruited were hospitalized in the Department of Gastroenterology of the Pomeranian Medical University in Szczecin. Final diagnosis of the disease was based on analysis of biopsy specimen, which in all cases revealed *adenocarcinoma* type of pancreatic malignancy. To establish staging of the disease, all patients underwent ultrasonography, computed tomography and/or endoscopic ultrasonography and chest x-ray examinations. Among the included individuals, six patients were qualified for surgical removal of the pancreatic tumour, eight patients presented inoperable locally advanced disease and 15 had distal metastases. Upon inclusion to the study, none of the patients was on chemotherapy treatment, received any cytotoxic agents/drugs within the last 12 months before the study, nor presented signs of an active infectious disease. General characteristics of the individuals enrolled in the study, together with statistical comparison of these features between examined groups, are presented in Table 1.

**Table 1:** General characteristic of surgical procedure and of individuals enrolled in the study (means ± S.D.)

	Control group	Pancreatic cancer patients
Age (years)	58 ± 11	61 ± 7
Gender (M-men/W-women)	(10-M/9-W)	(19-M/10-W)
BMI (kg/m^2^)	25.12 ± 3.43	26.18 ± 4.56
RBC (×10^12^ cells/l)	4.67 ± 0.73	4.38 ± 0.84
Hb (g/dl)	14.26 ± 1.57	12.56 ± 2.63
Platelets count (×10^9^ cells/l)	209 ± 47	253 ± 102
WBC count (×10^9^ cells/l)	8.01 ± 1.91	9.16 ± 4.09
CRP (mg/l)	3.07 ± 2.88	8.57 ± 2.14*
CA19.9 (U/ml)	17.81 ± 4.15	194.11 ± 73.01*
Subjective pain intensity (n [%])		
None	17 [89.5]	15 [51.7]
Delicate	2 [10.5]	6 [20.7]
Moderate	0 [0]	4 [13.8]
Intensive	0 [0]	4 [13.8]
General feeling (n [%])		
Good	19 [100]	15 [51.7]
Poor	0 [0]	10 [34.5]
Terrible/very weak	0 [0]	4 [13.8]

BMI: body mass index; RBC: red blood cells; Hb: haemoglobin; WBC: white blood cells; CRP: C-reactive protein.

* *P* < 0.01 (*versus* control group).

Peripheral blood samples (8–10 ml) were collected from all included individuals. The absolute numbers of leucocytes and lymphocytes in PB were determined at the same time with an automatic cell counter (SYSMEX XT-2000i; Sysmex Corporation, Kobe, Japan). Blood samples were centrifuged to obtain whole cell pellet and plasma fractions. Subsequently, plasma samples were frozen and stored at −80°C until further assessment of selected growth/inhibitory factors and immunomodulatory molecules. The population of PB-derived leucocytes was obtained from collected cell pellets after lysis of red blood cells with ammonium chloride-based lysing solution (BD Pharm Lyse Buffer; BD Biosciences Pharmingen, San Diego, CA, USA). Purified whole leucocyte factions were further used for staining and flow cytometric analysis towards stem/progenitor cell identification as described below. PB samples utilized for identification and isolation of SC populations were processed up to 12 hrs after blood draw from individual patients. The same processing and cell isolation procedures were applied to PB samples harvested from healthy and cancer individuals.

### Flow cytometry analysis of circulating populations of BMSCs

Flow cytometry analysis was performed according to the procedures previously described 15, 16. Briefly, circulating VSELs (FSC^low^/SSC^low^/CD45^−^/Lin^−^/CD133^+^ and FSC^low^/SSC^low^/CD45^−^/Lin^−^/CD34^+^ cells) and HSCs (CD45^+^/Lin^−^/CD133^+^ and CD45^+^/Lin^−^/CD34^+^ cells) were identified following immunostaining of the whole PB-derived nucleated cell fraction against haematopoietic lineage markers (Lin), CD45 antigen, CD133, or CD34. Antibodies for Lin markers included the following fluorescein isothiocyanate (FITC)-conjugated murine anti-human antibodies directed against following antigens: CD2, CD3, CD14, CD66b, CD24, CD56, CD16, CD19 and CD235a. EPCs (CD45^−^/CD31^+^/CD133^+^ and CD45^−^/CD31^+^/CD34^+^/KDR^+^ cells) were stained with fluorescent-labelled antibodies for CD45, CD31, CD133, CD34 and KDR (also known as VEGFR2), while the labelling of MSCs employed antibodies for such antigens as CD45, CD105 and Stro-1. Appropriate sets of isotype control antibodies were used for each staining and such negative control samples were used to set up gating strategy for identification of all indicated SC populations (VSELs, HSCs, EPCs and MSCs).

**Fig. 1:**
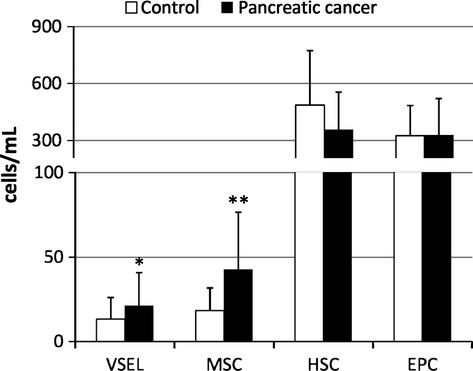
Results of cytometric analyses. Mean absolute numbers of stem/progenitor cells circulating in peripheral blood (PB) in control individuals and patients with pancreatic cancer together with their statistical comparison (means ± S.D.). VSEL: very small embryonic-like stem cells; MSC: mesenchymal stem cells; HSC: haematopoietic stem cells; EPC: endothelial progenitor cells. *P < 0.05; **P < 0.01 (level of significance vs control individuals).

In addition, a single-cell suspension was stained for lineage markers (CD56, CD235a, CD3, CD66b, CD24, CD19, CD14, CD16 and CD2) conjugated with fluorescein isothiocyanate, CD45 conjugated with PE and CXCR4 conjugated with APC. Samples were incubated with antibodies in PBS containing 2% foetal bovine serum (FBS; Life Technologies, Grand Island, NY, USA) for 30 min. on ice and then were washed and fixed with 4% paraformaldehyde solution for 20 min. Fixed cells were subsequently stained with Hoechst 33342 (2 μg/ml, Sigma-Aldrich, St. Louis, MO, USA) to visualize nucleated objects and exclude debris from subsequent analysis with an LSR II flow cytometer (Becton Dickinson, Franklin Lakes, NJ, USA). Gating strategy for selected SC populations based on negative controls is shown in Figure S1. The absolute numbers of circulating stem/progenitor cells/μl of PB were computed based on (*i*) the percentage content of each subpopulation within the whole leucocyte fraction and (*ii*) the white blood cell (WBC) count. The absolute numbers of circulating stem cells were then re-calculated per millilitre of PB (Fig. 1) and 1 × 10^6^ of leucocytes (Figure S2).

### ImageStream analysis of circulating BMSCs

Preparation of cells for ImageStream system (ISS) analysis was performed as described for classical flow cytometric methods 16. Briefly, the whole population of PB-derived leucocytes was subsequently stained for morphological characterization of circulating VSELs with fluorescent-labelled antibodies for haematopoietic lineage markers (Lin, FITC and the set of antibodies described above for flow cytometry), CD45 and CD34. Cells were further fixed with 4% paraformaldehyde solution for 20 min. at room temperature and nuclei were stained with Hoechst 33342 (2 μg/ml, Sigma-Aldrich). To identify the subpopulation of circulating VSELs expressing markers of pluripotency, such as Oct-4 and Nanog, the whole population of PB-derived leucocytes was initially fixed with 4% paraformaldehyde for 20 min., permeabilized with 0.1% Triton X-100 solution for 10 min., and subsequently stained for the nuclear transcription factors Oct-4 and Nanog with purified primary antibodies against Oct-4 (clone 7F9.2; Millipore, Billerica, MA, USA) and Nanog (clone N-17, cat. no. sc-30331; Santa Cruz Biotechnology, Santa-Cruz, Dallas, TX, USA), respectively. Staining was performed in PBS containing 2% FBS (Invitrogen). All samples were analysed with an ImageStream imaging cytometer system (Amnis Corp., Seattle, WA, USA).

### Analysis of systemic levels of complement cascade protein cleavage fragments, SDF-1, growth/inhibitory factors and S1P

The concentrations of SDF-1, VEGF, EGF, HGF, LIF, IGF and CC molecules (C3a, C5a, C5b-9/MAC) were measured using commercially available, high-sensitivity ELISA kits (R&D Systems, Minneapolis, MN, USA and BD Bioscience OptEIA ELISA Kits, MD, USA) according to the manufacturer's instructions. Extracellular haemoglobin (eHb) levels were determined using spectrophotometry according to previously described protocols 17, 18. To measure S1P concentrations, reverse-phase high-performance liquid chromatography (RP-HPLC) was employed 19. Briefly, specified volume of internal standard (S1P C17), 1 M NaCL and methanol was added to plasma, mixed, chloroform was added and mixture was again centrifuged. Lower organic phase was transferred to another tube. To the non-organic phase, chloroform was added and mixture was centrifuged. Two organic phases were combined, and dried in vacuum centrifuge. Dried substance was diluted in methanol, reactive mixture was added (*o-phthaldialdehyde, methanol, mercaptoethanol, boric acid pH 10.5*), incubated and finally centrifuged. Clear supernatant was transferred to fresh tube and analysed using high-performance liquid chromatography. Separation was performed using isocractic method and following separation conditions/tools were applied: Cosmosil 5 μm C18-ARII (150 × 4.6) column; cartridge 5 μm C18-ARII (10 × 4.6); moving phase: 10 mM K_2_HPO_4_: methanol: 1 M TBAP (78:21.5:0.5), temperature 25°C, flow 1.0 ml/min.; fluorescence detector (excitation: 340 nm; emission: 455 nm).

### Statistical methods

To determine the distribution of the continuous variables analysed, the Shapiro–Wilk's test was used. For comparison of mean parameter values between examined groups, Student's *t*-test was used (for normally distributed variables). For variables that were not normally distributed, the variable values were log transformed. If a normal distribution was then achieved, these transformed variables were also compared using Student's *t*-test. However, if the transformation did not create a normal distribution, the Mann–Whitney U-test was performed. Correlations between various analysed parameters were calculated using Pearson's test or Spearman's rank test, according to the normality of the distribution. Statistical analysis was performed using SPSS statistical analysis software, and significance was defined as *P* < 0.05.

The study protocol was approved by the Bioethical Committee of the Pomeranian Medical University in Szczecin, and patients provided informed written consent for participation.

## Results

### Analysis of included individuals

Initial comparison of the analysed groups of recruited individuals revealed significantly higher C-reactive protein (that still were relatively within reference range) and CA19.9 levels in patients with pancreatic malignancy (Table 1). No other significant differences were found between the analysed groups.

### Comparison of circulating stem cells' population in patients with pancreatic cancer and healthy individuals

Mean absolute numbers of various SCs populations circulating in PB in patients with pancreatic cancer and healthy individuals are depicted in Figure 1 and Figure S2. Our analysis demonstrated that, in comparison to healthy individuals, significantly higher numbers of circulating VSELs and MSCs are observed in patients with pancreatic malignancy (Fig. 1). Interestingly, numbers of other circulating stem cell populations, that is HSCs and EPCs, do not seem to be associated with the presence of malignancy, and levels of these BM-derived SCs seem to be slightly, although not significantly (*P* = 0.07 for HSCs and *P* = 0.14 for EPCs), lower than in control individuals. Unfortunately, when we divided our cancer patients into subgroups, according to the stage of the malignancy, we could not find any significant differences between the subgroups in absolute numbers of circulating SCs populations (Table S1).

### Potential involvement of the SDF-1, growth/inhibitory factors, complement molecules and S1P in mobilization of BMSCs into PB in patients with pancreatic malignancy

**Fig. 2:**
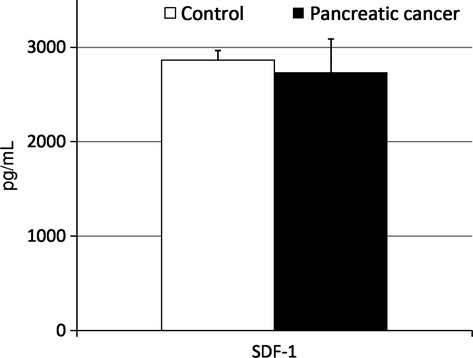
Mean systemic concentrations of stromal-derived factor-1 (SDF-1) in pancreatic cancer patients, together with their statistical comparison with values observed in healthy individuals (means ± S.D.). SDF-1: stromal-derived factor-1.

**Fig. 3:**
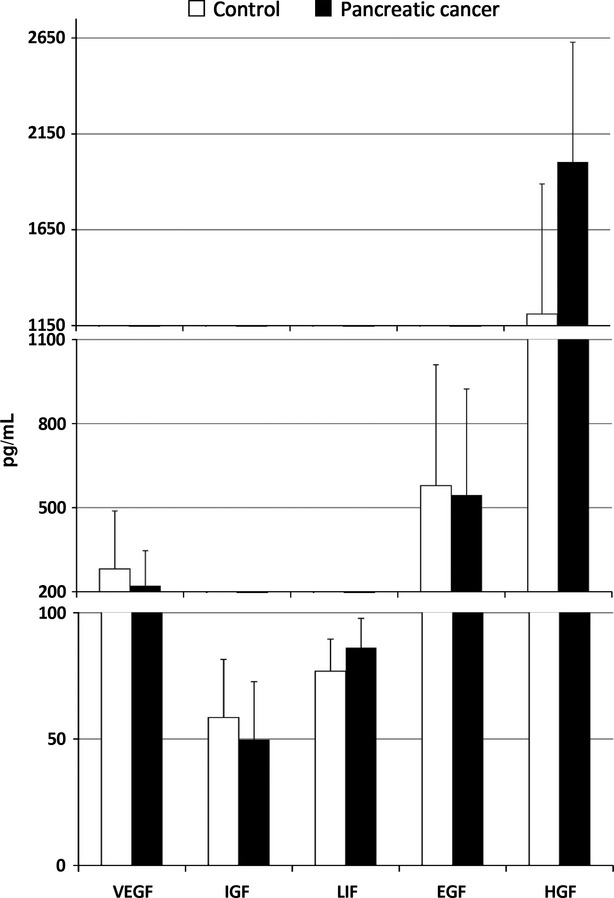
Mean systemic concentrations of growth/inhibitory factors in pancreatic cancer patients and healthy individuals, together with their statistical comparison between the groups (means ± S.D.). Statistical analysis revealed following levels of significance for comparison of mean values detected in healthy individuals and pancreatic cancer patients: *P* = 0.34 (for VEGF), *P* = 0.10 (for IGF), *P* < 0.05 (for LIF), *P* = 0.73 (for EGF) and *P* < 0.05 (for HGF). EGF: epidermal growth factor; HGF: hepatocyte growth factor; IGF: insulin-like growth factor; LIF: leukaemia inhibitory factor; VEGF: vascular/endothelial growth factor.

Our analysis within the groups demonstrated that in generally healthy controls, absolute numbers of circulating stem cells in PB, such as MSCs or EPC, are associated with some anthropometric features of examined individuals, such as BMI and age (*r* = −0.90; *P* < 0.03 and *r* = −0.75; *P* < 0.05 respectively). Interestingly, in patients with pancreatic malignancy, we could not demonstrate any significant correlation between analysed anthropometric parameters and absolute numbers of circulating SCs. To establish whether observed peripheral increase in numbers of circulating SCs is associated with systemic changes in concentrations of well-known chemoattractant (SDF-1) and/or growth/inhibitory factors, we measured levels of these molecules in both analysed groups (Figs 2 and 3). Results demonstrated that patients suffering from pancreatic malignancy have slightly, although not significantly (*P* = 0.22), lower mean SDF-1 level than healthy individuals. While we could establish various significant correlations between absolute numbers of selected populations of circulating SCs and SDF-1 levels in healthy individuals [with VSELs (*r* = 0.34) and HSCs (*r* = 0.25), *P* < 0.05 for both], in patients with pancreatic malignancy such associations were not observed, as well as, SDF-1 levels were comparable between patients suffering from resectable, locally advanced and metastatic disease (Table S1). In addition, no statistically significant differences in mean levels of VEGF, IGF and EGF were observed between healthy individuals and cancer patients. Levels of HGF and LIF were significantly higher in patients with pancreatic malignancy (Fig. 3). However, in our study, only the systemic HGF levels were correlating with absolute numbers of circulating MSCs (*r* = 0.72, *P* < 0.03), but not with VSELs (*r* = 0.21, *P* = 0.28) in patients with malignancy.

**Fig. 4:**
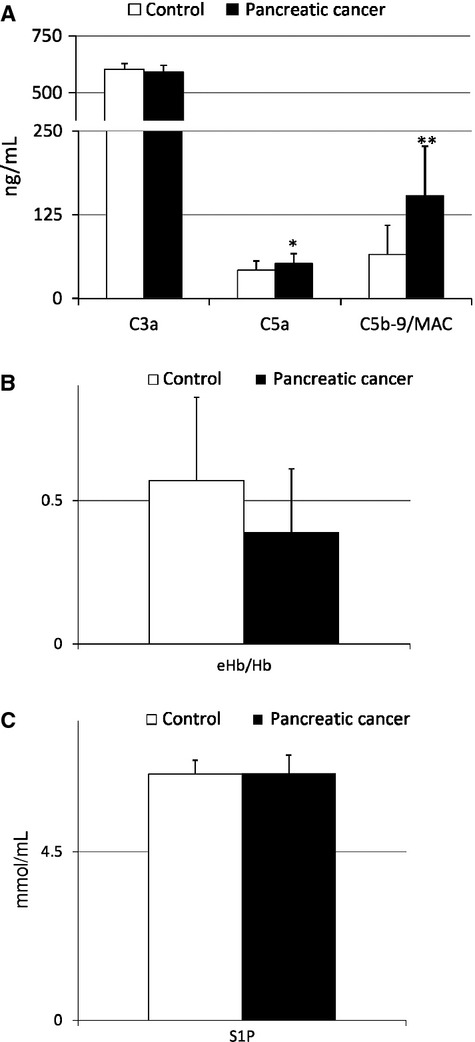
Mean concentrations of examined complement anaphylatoxins/molecules (**a**), relative eHb values^#^ (**b**), and S1P (**c**) in pancreatic cancer patients and healthy individuals, together with their statistical comparison between analysed groups (means ± S.D.). MAC: membrane attack complex; eHb: extracellular haemoglobin; S1P: sphingosine-1-phosphate. *P < 0.05; **P < 0.005 (level of significance vs control individuals). # values calculated as a coefficient derived from a formula: eHb = direct eHb level/plasma Hb level.

We also wanted to verify whether activation of CC (C3a, C5a and C5b-9/MAC) may be associated with increased numbers of circulating BMSCs, that are observed in patients with pancreatic cancer. In our study, patients suffering from pancreatic malignancy had significantly higher systemic levels of C5a and C5b-9/MAC molecules, while C3a concentrations were statistically comparable with those observed in healthy individuals (Fig. 4a). Interestingly, in our study, levels of selected examined complement fractions were strongly and significantly correlating with absolute numbers of circulating VSELs and MSCs in patients with pancreatic cancer (Table 2), while such tendencies could not be detected in the control group. Of note, we already reported that activation of distal part of CC and release of C5a and C5b-9/MAC is essential for optimal egress of SCs from BM 20.

**Table 2:** Coefficients of correlations between absolute numbers of circulating stem cells' populations and systemic levels of complement anaphylatoxins measured in patients with pancreatic cancer

Stem cells population/parameter	C3a	C5a	MAC
Pancreatic cancer patients			
VSEL	−0.70#	0.83*	0.75#
MSC	NS	−0.91*	−0.67#
HSC	NS	NS	NS
EPC	NS	NS	NS

P: level of significance; NS: not significant; VSEL: very small embryonic-like stem cells; MSC: mesenchymal stem cells; HSC: haematopoietic stem cells; EPC: endothelial progenitor cells; MAC: membrane attack complex (C5b-9) of the complement system.

# *P* < 0.05.

* *P* < 0.005.

Finally, we decided to analyse whether observed C5b-9/MAC levels are associated with erythrocytes' lysis (expressed by levels of eHb concentrations), and with increase in plasma S1P levels in healthy and pancreatic cancer patients. However, in our study, we could not determine any significant correlation between C5b-9/MAC levels and eHb concentration in plasma, which were comparable between both examined groups (*P* = 0.49; Fig. 4b). Furthermore, systemic levels of S1P were found to be comparable between the analysed groups (*P* = 0.73), as well as between patients suffering from resectable, locally advanced and metastatic disease (Fig. 4c and Table S1). However, only in pancreatic cancer patients, S1P plasma concentrations were significantly correlated with the absolute numbers of circulating PB VSELs, MSCs and HSCs (*r* = 0.59, −0.63 and 0.71, respectively, *P* < 0.01 for all), as well as, with systemic SDF-1 level (*r* = 0.85, *P* = 0.004).

## Discussion

Recently, there has been heightened interest in the potential involvement of normal SCs in the development of malignancies. Even though in several experimental animal studies various researchers preliminarily demonstrated that SCs may contribute to a development of GI cancers 6, 7, still precise mechanisms regulating this phenomenon are neither not known nor examined in the clinical setting. Therefore, in this study, we have comprehensively analysed circulating numbers of various BMSCs populations in patients suffering from pancreatic cancer, as well as, measured several soluble factors that are responsible for BMSCs mobilization/retention.

In our study, we have found that pancreatic malignancy in human beings is associated with increased numbers of systemically circulating BM-derived Lin^−^/CD45^−^/CD133^+^ and CD45^−^/CD105^+^/STRO-1^+^ cells enriched for VSELs and MSCs, respectively, that are BMSCs populations that possess molecular potential for trans-differentiation into or regeneration of solid organs. We hypothesize that these circulating BMSCs populations are mobilized in the course of pancreatic cancer development to support its growth and spread. Our hypothesis may also be supported by results of the previous studies, in which it was demonstrated that BMSCs migrate to murine pancreatic tissue for promotion of its regeneration, as well as, human pancreatic tumours are mainly created by cancer SCs possessing high expression of CD133 antigen and embryonic markers such as Oct-4 21–23. Interestingly, these markers are expressed by the population of circulating BM-derived VSELs examined in our study. Furthermore, in our study, we observed somehow ‘selective’ egress/mobilization of BMSCs into PB – as only SCs populations that participate in solid organ/tissues regeneration were mobilized. In contrast, HSCs or EPCs did not seem to become mobilized in our patients during development of pancreatic malignancy.

Moreover, our study also demonstrated that egress of BMSCs in patients with pancreatic cancer seems to be executed in an SDF-1-independent manner. While in several studies, it has been reported that during development of pancreatic cancer the expression of SDF-1 increases within pancreatic tissue 24, 25, results of our study demonstrate that SDF-1 does not seem to be associated with intensified peripheral trafficking of BMSCs in patients with pancreatic cancer and its systemic concentrations are comparable to those observed in healthy individuals. Similar results have also been recently observed by Vizio and colleagues 26. This discrepancy may be explained by the fact of enhanced activity of SDF-1 degrading proteolytic enzymes in plasma (such as matrix metalloproteinase-9) that is observed in majority of patients 27. In contrast to plasma SDF-1 level, we noticed strong associations between CC cleavage fragments and increased BMSCs trafficking. To support this, systemic levels of C5a and C5b-9/MAC were not only elevated, but also strongly correlated with the increased absolute numbers of circulating BMSCs in patients with pancreatic cancer. Our results are therefore in agreement with some previous animal studies, which highlighted crucial involvement of the complement cascade in promotion of BMSCs egress from the BM environment 13. In addition, our findings also seem to indirectly support and translate previous experimental observations that selected complement anaphylatoxins are likely to possess strong chemoattractive properties for systemic migration of SCs during development of malignancy in human beings 28.

Finally, even though it has been reported that S1P is a major chemoattractant responsible for egress of HSCs from the BM, and reported that its levels following HSCs mobilization increase in experimental animals 13, 14, in our clinical studies such drastic changes in systemic S1P levels in patients with pancreatic cancer were not observed. This suggests that S1P may not play a crucial role in egress of non-HSCs (MSCs and VSELs) from BM into PB in patients with growing pancreatic tumour. On the other hand, we and others have demonstrated that systemic increase in S1P levels is not essential for BMSCs mobilization in patients, as basal S1P concentrations are already sufficient to facilitate BMSCs egress from the BM when retentive factors are suppressed 13, 29. Nevertheless, further studies are undoubtedly needed to clarify the exact nature of this association between BMSCs mobilization and S1P activity during development of pancreatic cancer in human beings.

We are aware that we have described herein an interesting phenomenon and further studies are needed to explain what is its clinical/pathological significance. It would be interesting to investigate if similar phenomenon also occurs in other types of gastrointestinal malignancies, as well as to address a basic issue – what is the role of these SCs mobilized into circulation during pancreatic cancer development. Do they contribute to expansion of growing tumour by providing vasculature and stroma or just it is a kind of overall response of organism to tissue organ injury and hypoxic microenvironment of expanding tumour 12.

In summary, our study demonstrated that in patients with pancreatic cancer, intensified peripheral trafficking of BMSCs occurs. This phenomenon does not seem to be caused by systemic SDF-1 concentrations, but rather is associated with increased levels of complement molecules (mainly C5a and C5b-9) and HGF. Finally, our study highlights several significant associations between systemic HGF, S1P levels and enhanced egress of BMSCs in patients with pancreatic cancer.

## Supporting information

**Table S1.** Results of cytometric and biochemical analyses in pancreatic cancer patients, subdivided into groups according to the advancement of malignancy (presented as means ± S.D. or medians [interquartile range]).

**Figure S1**. Representative flow cytometric analysis of VSELs and HSCs circulating in human peripheral blood of cancer patients.

**Figure S2.** Results of cytometric analyses – absolute numbers per 1 million of WBC.
