# Supplementary material for: An intensified systemic trafficking of bone marrow-derived stem/progenitor cells in patients with pancreatic cancer
Source: J Cell Mol Med. 2013 May 15;17(6):792–9. doi: 10.1111/jcmm.12065 (PMC3823183; doi:10.1111/jcmm.12065)
Supplement: Supplementary file 1 [file jcmm0017-0792-SD1.pdf]

Supplementary Table 1. Results of cytometric and biochemical analyses in pancreatic cancer patients, subdivided into groups according to the advancement of malignancy (presented as means ± SD or medians [interquartile range]).

**Subgroup/ Parameter**	**Early/Resectable (n = 6)**	**Locally advanced (n = 8)**	**Distal metastases (n = 15)**
Cytometric analyses			
VSEL (cells/mL)	23 [18,28]	24 [13,35]	26 ± 13
MSC (cells/mL)	40 [25,55]	39 [26,52]	42 ± 22
HSC (cells/mL)	308 [108,508]	300 [111,489]	335 ± 226
EPC (cells/mL)	306 [131,481]	318 [125,511]	315 ± 184
Biochemical analyses			
SDF-1(pg/mL)	2871.11 [2672.95; 3069.27]	2903.31 [2685.47; 3121.15]	2850.0 ± 293.15
VEGF (pg/mL)	227.43 [127.39; 327.47]	231.98 [114.72; 349.25]	218.84 ± 128.64
IGF (pg/mL)	50.04 [37.27; 62.81]	46.75 [22.84; 70.66]	48.99 ± 28.17
LIF (pg/mL)	82.83 [69.96; 95.70]	85.02 [71.01; 99.03]	84.77 ± 13.83
EGF (pg/mL)	528.96 [123.08; 934.84]	514.99 [132.84; 897.14]	520.84 ± 418.75
HGF (pg/mL)	1938.21 [1422.88; 2453.54]	2000.08 [1410.92; 2589.24]	1901.17 ± 748.03
C3a (ng/mL)	598.35 [579.33; 617.37]	600.12 [581.19; 619.05]	590.0 ± 29.45
C5a (ng/mL)	52.78 [39.78; 65.78]	50.0 [42.01; 57.99]	51.28 ± 12.83
C5b-9/MAC (ng/mL)	151.48 [89.3; 213.66]	158.37 [98.28; 218.46]	153.85 ± 73.92
eHb	0.33 [0.11; 0.55]	0.33 [0.22; 0.54]	0.39 ± 0.22
S1P (mmol/mL)	6.53 [6.08; 6.99]	6.54 [6.12; 6.96]	6.60 ± 0.49

VSEL – very small embryonic-like stem cells

MSC – mesenchymal stem cells

HSC – hematopoietic stem cells

EPC – endothelial progenitor cells

SDF-1 – stromal-derived factor-1

VEGF – vascular/endothelial growth factor

EGF – epidermal growth factor

HGF – hepatocyte growth factor

IGF – insulin-like growth factor

LIF – leukemia inhibitory factor

MAC – membrane attack complex

eHb – extracellular hemoglobin

S1P – sphingosine-1-phosphate

Supplementary Figure 1. Representative flow cytometric analysis of VSELs and HSCs circulating in human peripheral blood of cancer patients.

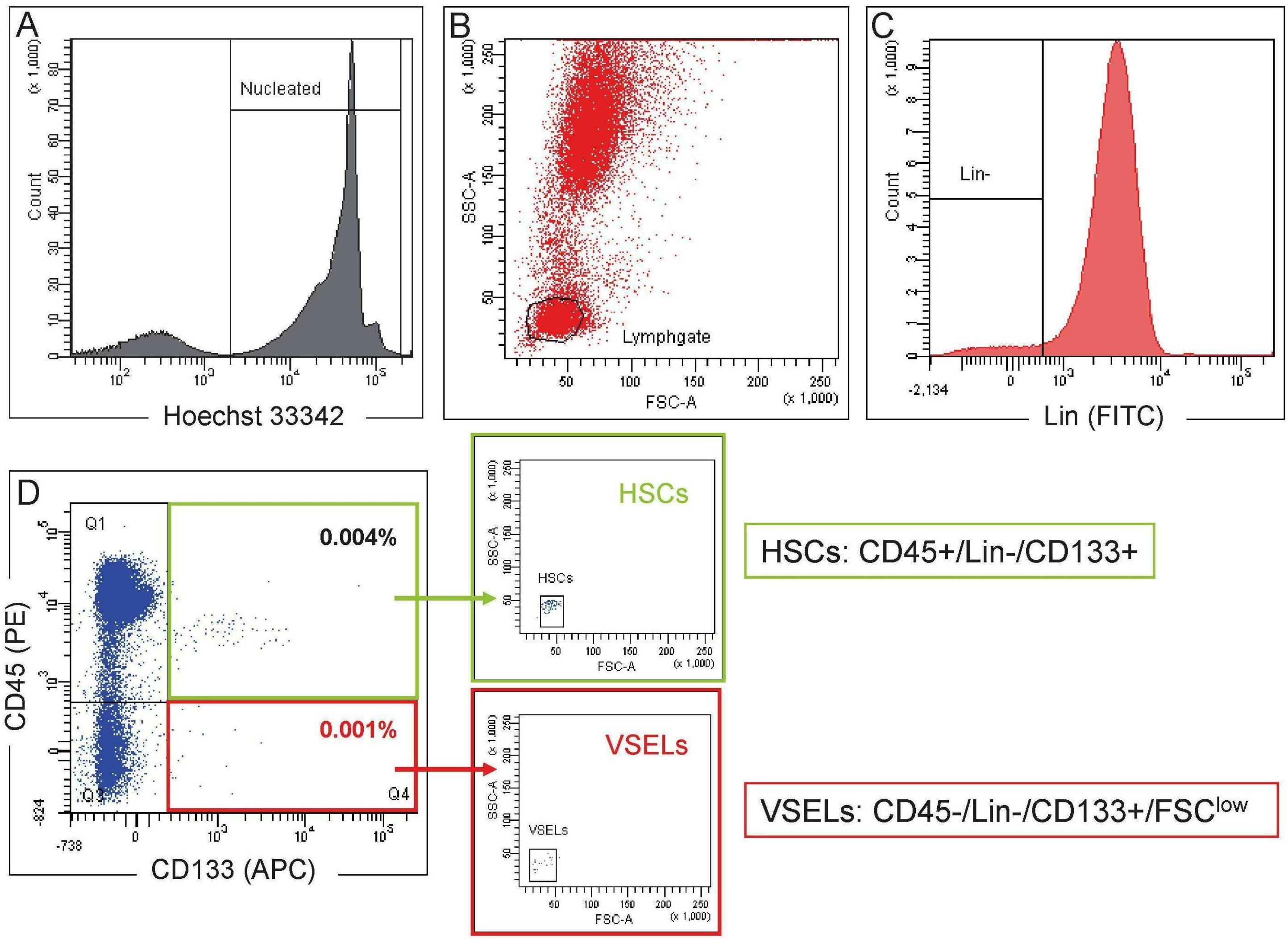

Graphs show histograms and dot-plots presenting gating approach including: identification of nucleated cells following staining of fixed leukocytes with Hoechst 33342 (Panel A), gating for lymphocytes extended into fraction of small objects - less than 5 mm (Panel B), and identification of cells negative for hematopoietic lineages markers (Panel C). Panel D shows analysis of cells from gate “Lin-“ regarding to CD45 vs. CD133 expression. Hematopoietic stem/progenitor cells (HSCs) are identified as CD45+/Lin-/CD133+ cells, while VSELs are CD45-/Lin-/CD133+. Back-gating of indicated subpopulations of VSELs and HSCs on FSC vs. SSC plots, show very small size of VSELs when compared with larger HSCs. Percentages represents average content of each population among all nucleated PB- derived cells.

Supplementary Figure 2. Results of cytometric analyses – absolute numbers per 1 mln of WBC.

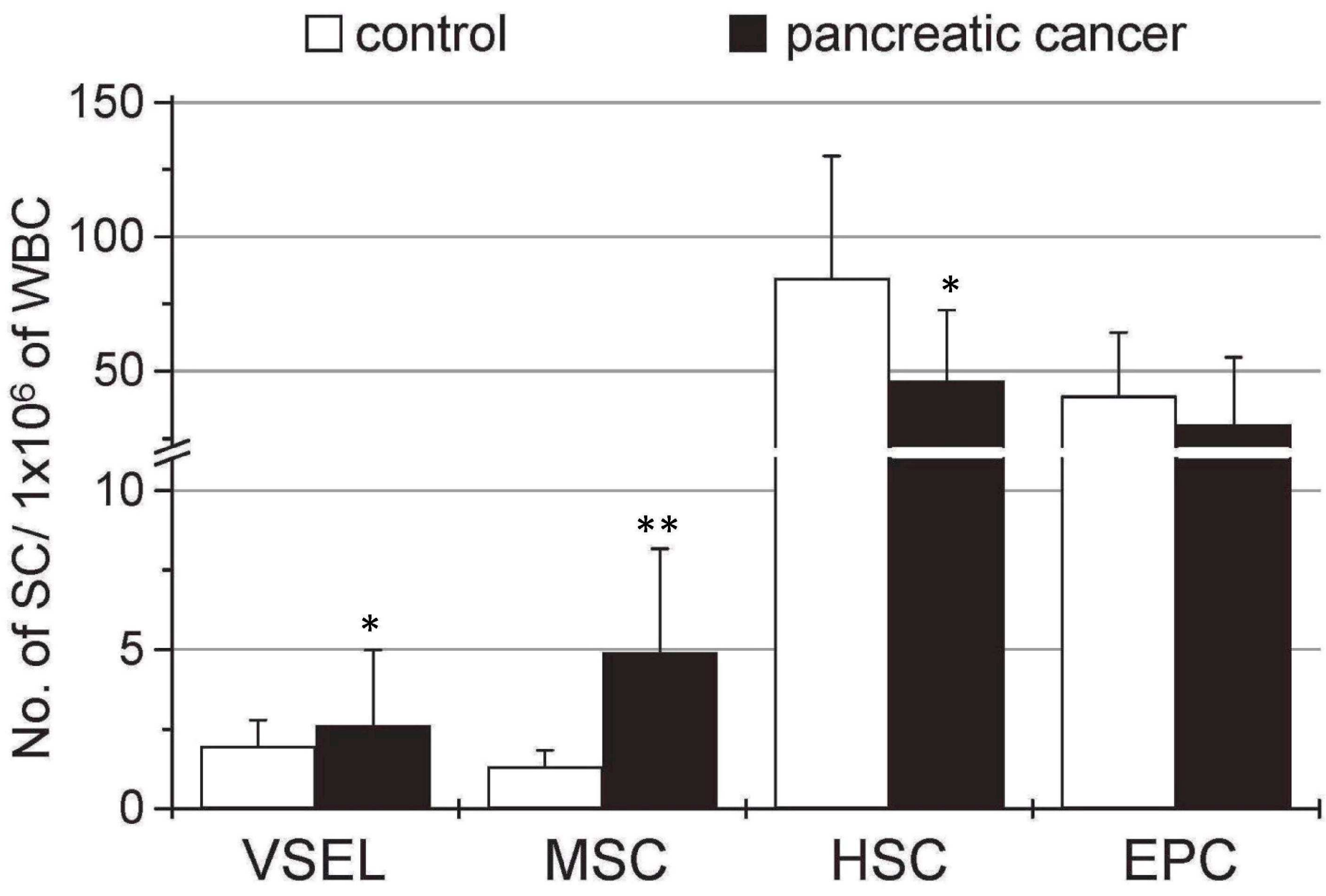

Mean absolute numbers of circulating stem/progenitor cells per $1x10^6$ of PB – derived leukocytes in control individuals and patients with pancreatic cancer together with their statistical comparison (means ± standard deviation).

VSEL – very small embryonic-like stem cells MSC – mesenchymal stem cells

HSC – hematopoietic stem cells EPC – endothelial progenitor cells

* P<0.05; ** P<0.01 (level of significance vs control individuals)
